# The effect of tobacco, XPC, ERCC2 and ERCC5 genetic variants in bladder cancer development

**DOI:** 10.1186/1471-2407-11-101

**Published:** 2011-03-22

**Authors:** Kamel Rouissi, Islem Ben Bahria, Karim Bougatef, Raja Marrakchi, Nejla Stambouli, Khouloud Hamdi, Mohamed Cherif, Mohamed Riadh Ben Slama, Mohamed Sfaxi, Fethi Ben Othman, Mohamed Chebil, Amel Benammar Elgaaied, Slah Ouerhani

**Affiliations:** 1 Laboratory of Genetic, Immunology and Human Pathology, Faculty of Sciences of Tunis, University of El Manar I. Tunis, Tunisia; 2 Department of Urology, Charles Nicolle Hospital, Tunis, Tunisia; 3 Laboratory of Molecular and Cellular Haematology, Pasteur Institute of Tunis, University of Tunis El Manar, Tunis, Tunisia

**Keywords:** Gene repair, Bladder cancer, Polymorphism, Tunisia

## Abstract

**Background:**

In this work, we have conducted a case-control study in order to assess the effect of tobacco and three genetic polymorphisms in *XPC, ERCC2 and ERCC5 *genes (rs2228001, rs13181 and rs17655) in bladder cancer development in Tunisia. We have also tried to evaluate whether these variants affect the bladder tumor stage and grade.

**Methods:**

The patients group was constituted of 193 newly diagnosed cases of bladder tumors. The controls group was constituted of non-related healthy subjects. The rs2228001, rs13181 and rs17655 polymorphisms were genotyped using a polymerase chain reaction-restriction fragment length polymorphism technique.

**Results:**

Our data have reported that non smoker and light smoker patients (1-19PY) are protected against bladder cancer development. Moreover, light smokers have less risk for developing advanced tumors stage. When we investigated the effect of genetic polymorphisms in bladder cancer development we have found that ERCC2 and ERCC5 variants were not implicated in the bladder cancer occurrence. However, the mutated homozygous genotype for XPC gene was associated with 2.09-fold increased risk of developing bladder cancer compared to the control carrying the wild genotype (p = 0.03, OR = 2.09, CI 95% 1.09-3.99). Finally, we have found that the XPC, ERCC2 and ERCC5 variants don't affect the tumors stage and grade.

**Conclusion:**

These results suggest that the mutated homozygous genotype for XPC gene was associated with increased risk of developing bladder. However we have found no association between rs2228001, rs13181 and rs17655 polymorphisms and tumors stage and grade.

## Background

Bladder cancer incidence and mortality rates vary about 10-fold worldwide [1,2]. The highest rates are found in North America and Western Europe, and are lower in Eastern Europe and several parts of Asia [1]. Bladder cancer is a smoking-related cancer [3]. Urothelial cell carcinomas (UCC) represent more than 90% of bladder tumors and are classified into superficial (pTa and pT1) and muscle invasive (≥ pT2) stages. The majority of superficial tumors recur but progression to muscle invasion is relatively infrequent. Only high-grade superficial tumors (pTa GIII and pT1 GIII) progress to invasive disease and represent a high-risk for death from disease [4].

Cigarette smoking is the predominant risk factor for bladder cancer and is estimated to be responsible for 50% of the cases in men, and 30% of the cases in women [3]. Cigarette smoke is a rich source of reactive oxygen species that can induce a variety of DNA damage. The body's two primary defence mechanisms against mutagenic exposure are DNA damage repair systems and metabolic enzyme checkpoints [5]. Using these two mechanisms facilitates cellular responses to DNA damage from endogenous and exogenous mutagenic exposures to maintain genomic integrity [5,6]. There are four major DNA repair pathways in human cells: mismatch repair (MMR), nucleotide-excision repair (NER), base-excision repair (BER), and double-strand break (DSB) repair [6]. The damage caused by cigarette smoke is mainly removed by the nucleotide- excision repair pathway, and to a lesser extent, the base-excision repair pathway [7]. The NER pathway mainly removes bulky DNA adducts typically generated from exposure to polycyclic aromatic hydrocarbons present in tobacco smoke. The BER pathway is responsible for removal of oxidized DNA bases that may arise endogenously or from exogenous agents [8].

The NER pathway has been reported to be the most significant modulator of bladder cancer risk and implicated many enzymes such as Xeroderma pigmentosum type C, D and G (XPC, ERCC2 and ERCC5). The XPC enzyme is an important DNA damage recognition protein that binds to damaged DNA at a very early stage during DNA repair [9]. The ERCC5 (xeroderma pigmentosum type G) protein is essential for the two incision steps in nucleotide-excision repair [10]. The ERCC2 (xeroderma pigmentosum type D) protein takes part in the nucleotide excision repair pathway, which recognizes and repairs a wide range of structurally unrelated lesions such as bulky adducts and thymidine dimers. This protein works as an ATP-dependent (5'--->3') helicase joined to the basal TFIIH complex to separate double helix [11].

Many polymorphisms were detected in XPC, ERCC2 and ERCC5 genes and alter the ability of the encoded enzymes to repair the DNA damage. These variants may increase susceptibility to bladder cancer through complex gene-gene and gene-smoking interactions [12]. Among variants which were studied in association with bladder cancer we note Lys939Gln genotype (A > C; rs2228001) in XPC gene, Lys751Gln (rs13181) in ERCC2 gene and Asp1104His (G > C; rs17655) in ERCC5 gene. Recently; many reported case-control studies have analyzed the association between risk factors (tobacco and NER genes polymorphisms) and bladder cancer development. However these studies have reported conflicting results [12-17]. To the best of our knowledge, no study has been done in Tunisia on the association among these 3 polymorphisms and the risk of bladder cancer. We hypothesized that these 3 polymorphisms in three genes might contribute to the etiology of Bladder cancer. To test this hypothesis, we genotyped Lys939Gln genotype (A > C; rs2228001), ERCC2 Lys751Gln (rs13181) and ERCC5 Asp1104His (G > C; rs17655) polymorphisms in our ongoing hospital-based case-control study of Bladder cancer in a population from Tunisia. We have also try to evaluate whether these studied single nucleotide polymorphisms (SNP) affect tumors phenotypes by investigating associations between the SNP and tumors stage and grade.

## Methods

### Subjects

This study was approved by a local ethical committee. After giving informed consent, the demographic details were obtained by interviewing each subject and peripheral blood samples were collected from all subjects into tubes with EDTA at pH 8. A total of 193 patients with urothelial cell carcinoma of bladder cancer and 193 control subjects were included in the present study. The controls were recruited daily from patients newly diagnosed and treated at the same urology department for benign diseases, mainly prostatic hyperplasia, cystitis and urolithiasis. Patients with cancer, or liver or renal diseases, were excluded. This control group was constituted of non-related subjects who were matched to the case group for sex proportion, geographic origin, and age range. Patients were recruited from the Department of Urology at the Charles Nicole Hospital in Tunisia. All were from the North of Tunisia, 91.2% of them were men, and the mean age at diagnosis was 65.23 ±11.3 years. These patients were classified according to their tobacco status. The smoker category included current smokers who smoked daily. Non consumers of tobacco were defined as persons who had never smoked or had consumed less than 20 packs of cigarettes or 360 g of tobacco in their lifetime or less than one cigarette per day. The intensity of tobacco use (PY) was defined as the amount of tobacco consumed during the life of patients (1 PY = 7300 cigarettes smoked during 1 year). It was found that 79.79% (154/193) of patients were current smokers, and 20.21% were non-tobacco consumers. The clinical characteristics, including tumor stage and grade, were obtained from the urologist of our department. Tumors were staged according to the criteria of the tumor-node-metastasis classification (TNM) and the WHO-International Society of Urological Pathology as follows: 13 carcinoma in Situ (CIS), 34 pTa GI, 12 pTa GII, 3 pTa GIII, 53 pT1 GII, 34 pT1 GIII and 44 invasive tumors (≥ pT2).

### DNA preparation and genotyping

Genomic DNA was extracted from leukocytes using a phenol-chloroform procedure [18]. The quality of genomic DNA was controlled by electrophoresis on a 1% agarose gel stained with ethidium bromide. The XPC, ERCC2 and ERCC5 polymorphisms were detected with polymerase chain reaction-restriction fragment length polymorphism-based approaches (PCR-RFLP), as described previously [19]. The studied polymorphisms and details of RFLPs are shown in Table 1.

**Table 1:** Details of RFLPs studied

Gene/exon	Polymorphism (amino acid change)	SNP reference	Genotype	PCR product sizes (Pb)	Restriction enzyme
**XPC exon 16**	**A > C (Lys939Gln)**	rs2228001	**AA**		
			**AC**	**244**	**PvuII**
			**CC**		
**ERCC2 exon 23**	**A > C (Lys751Gln)**	rs13181	**AA**		
			**AC**	**348**	**PstI**
			**CC**		
**ERCC5 exon 15**	**G > C (Asp1104His)**	rs17655	**GG**		
			**GC**	**158**	**Hsp92II**
			**CC**		

### Statistical analysis

Departures from Hardy-Weinberg equilibrium were tested using the software package Arlequin ver 3.01 [20]. We have used Epi info 6.0 software to calculate the odds ratios (OR) value with 95% confidence intervals (CI). We have also used the logistic regression test of SPSS 16.0 software to investigate the impact of smoking and polymorphisms in repair genes on the development and progression of bladder cancer. The logistic regression is a mathematical modelling approach that is used to describe the relationship of several predictor variables X1, X2,..., Xk to a dichotomous dependent variable Y which is typically coded as 1 or 0 for its two possible categories.

## Results

Genotype and gene distributions for ERCC2, XPC and ERCC5 in 193 bladder cancer cases and 193 controls are summarized in Table 2. All samples were found to be in Hardy-Weinberg equilibrium, except for cases of XPC A > C polymorphism, among whom the genotypic distribution showed statistical differences from Hardy-Weinberg expectations (P = 0.008).

**Table 2:** Comparisons of the *XPC*, *ERCC2 *and *ERCC5 *alleles and genotypes distributions between patients and controls

	N (%)			
			
Alleles and Genotypes	Controls (N = 193)	Patients (N = 193)	P value	OR (95% CI)
*XPC *(rs2228001)				
AA	79 (40.9)	74 (38.3)	-	1*
AC	92 (47.7)	76 (39.4)	0.65	-
CC	22 (11.4)	43 (22.3)	0.04**	2.09 (1.09-3.99)
A	250 (64.8)	224 (58.0)	-	1*
C	136 (35.2)	162 (42.0)	0.06	-

*ERCC2 *(rs13181)				
AA	86 (44.6)	97 (50.3)	-	1*
AC	86 (44.6)	76 (39.4)	0.30	-
CC	21 (10.9)	20 (10.4)	0.75	-
A	258 (66.8)	270 (69.9)	-	1*
C	128 (33.2)	116 (30.1)	0.39	-

*ERCC5 *(rs17655)				
GG	87 (45.1)	95 (49.2)	-	1*
GC	86 (44.6)	70 (36.3)	0.21	-
CC	20 (10.4)	28 (14.5)	0.55	-
G	260 (67.3)	260 (67.3)	-	1*
C	126 (32.7)	126 (32.7)	0.93	-

95% CI: Confidence Interval, 1*: reference group, **: Corrected p value (Bonferroni's correction)

### Effect of tobacco and genetic polymorphisms of XPC, ERCC2 and ERCC5 in bladder cancer development

By using logistic regression test we have found that non smokers and light smokers (1-19PY) were protected against bladder cancer development (Table 3). The XPC*C, ERCC2*C and ERCC5*C variants were respectively detected at 35.23%, 33.16% and 32.64% of controls group. The comparison of patients and controls according to the frequencies of ERCC2 and ERCC5 genotypes did not show a significant statistic difference (Table 2). However, significant differences in genotypic frequencies were detected for the XPC A > C polymorphism. Indeed, the XPC C/C genotype was found to be at a significant 2.09-fold significant increased risk of developing bladder cancer compared to the control carrying the wild genotype (Table 2). In fact, the logistic regression casts a protective effect of XPC AA and AC genotype against bladder cancer development (Table 3).

**Table 3:** Case-control study: implication of tobacco and polymorphisms in *XPC*, *ERCC2 *and *ERCC5 *on bladder cancer development

								95% Confidence Interval for Exp(B)	
**group^a^**		**B**	**Standard error**	**Wald**	**Df**	**P value**	**Exp (B) = OR**	**Lower Bound**	**Upper Bound**

Cases	Intercept	-1,10	1,08	1,03	1	,31			
	Sex	,30	,42	,53	1	,46	1,36	,59	3,11
	Age range	1,82	,39	21,99	1	,00	6,22	2,89	13,35
	[XPC (A/C) = AA]	-,85	,34	6,06	1	,01	,42	,21	,84
	[XPC (A/C) = AC]	-1,01	,34	8,80	1	,00	,36	,18	,70
	[XPC (A/C) = CC]	0^b^	.	.	0	.	.	.	.
	[ERCC2 (A/C) = AA]	,12	,40	,10	1	,75	1,13	,51	2,51
	[ERCC2 (A/C) = AC]	-,16	,40	,15	1	,69	,85	,38	1,90
	[ERCC2 (A/C) = CC]	0^b^	.	.	0	.	.	.	.
	[ERCC5 (G/C) = CC]	,55	,38	2,11	1	,14	1,74	,82	3,71
	[ERCC5 (G/C) = GC]	-,22	,24	,85	1	,35	,79	,49	1,29
	[ERCC5 (G/C) = GG]	0^b^	.	.	0	.	.	.	.
	[Tobacco PY = 0]	-1,18	,32	13,03	1	,00	,30	,16	,58
	[Tobacco PY = 1-19]	-1,97	,45	19,17	1	,00	,13	,05	,33
	[Tobacco PY ≥ 20]	0^b^	.	.	0	.	.	.	.

This reduced model was obtained after adjustment to sex and age; a: The reference category is controls group; b: this parameter is set to zero because it is redundant; PY: pack years. Logistic regression: Number of observation = 386, Chi-square: 88.409, p = 0.000, Pseudo R-square = 0.205, Log likelihood = 206,541

### Effect of tobacco, XPC, ERCC2 and ERCC5 polymorphisms on tumors stage and grade

Tumors were staged according to the criteria of the tumor-node-metastasis classification (TNM) and the WHO-International Society of Urological Pathology as follows: 49 pTa, 87 pT1 and 44 invasive tumors (≥ pT2). The comparison of patients with advanced tumors stages to the reference group (pTa) according to genetic polymorphisms dose not show a significant difference (Table 4). The same result was obtained when we studied the effect of genetic polymorphisms on the grade of pT1 tumors group (Table 5). However, we have reported that the risk of developing pT1 tumors decrease significantly on light smokers (p = 0.03, OR = 0.15; CI 95% = 0.027-0.882). We have also found that non-smoker patients with pT1 have at a 6.88-fold significant increased risk of developing a GIII grade compared to the reference group (Table 5).

**Table 4:** Logistic regression: effect of tobacco and *XPC*, *ERCC2 *and *ERCC5 *polymorphisms on tumors stages

								95% Confidence Interval for Exp(B)	
								
stade ^a^		B	Std. Error	Wald	Df	**Sig**.	Exp(B)	Lower Bound	Upper Bound
Pt1	Intercept	-,55	1,80	,09	1	,75			
	Sex	,97	,78	1,54	1	,21	2,64	,57	12,20
	Age	-1,16	,47	6,06	1	,01	,31	,12	,78
	[XPC (A/C) = AA]	-,11	,53	,04	1	,82	,89	,31	2,53
	[XPC (A/C) = AC]	,49	,54	,83	1	,36	1,64	,56	4,74
	[XPC (A/C) = CC]	0^b^	.	.	0	.	.	.	.
	[ERCC2 (A/C) = AA]	,66	,64	1,07	1	,30	1,94	,55	6,84
	[ERCC2 (A/C) = AC]	,24	,65	,13	1	,71	1,27	,35	4,56
	[ERCC2 (A/C) = CC]	0^b^	.	.	0	.	.	.	.
	[ERCC5 (G/C) = CC]	,64	,65	,99	1	,31	1,91	,53	6,82
	[ERCC5(G/C) = GC]	,25	,41	,36	1	,54	1,28	,57	2,87
	[ERCC5(G/C) = GG]	0^b^	.	.	0	.	.	.	.
	[PY = 0]	,20	,56	,13	1	,71	1,22	,40	3,72
	[PY = 1-19]	-1,87	,89	4,41	1	,03	,15	,02	,88
	[PY ≥ 20]	0^b^	.	.	0	.	.	.	.

≥pT2	Intercept	-,62	3,04	,04	1	,83			
	Sex	1,27	1,41	,81	1	,36	3,58	,22	57,00
	Age	-1,71	,63	7,22	1	,00	,18	,05	,63
	[XPC(A/C) = AA]	-,42	,59	,51	1	,47	,65	,20	2,10
	[XPC(A/C) = AC]	-,19	,62	,09	1	,75	,82	,24	2,77
	[XPC(A/C) = CC]	0^b^	.	.	0	.	.	.	.
	[ERCC2(A/C) = AA]	,65	,80	,67	1	,41	1,93	,40	9,29
	[ERCC2(A/C) = AC]	,34	,80	,18	1	,67	1,40	,29	6,81
	[ERCC2(A/C) = CC]	0^b^	.	.	0	.	.	.	.
	[ERCC5(G/C) = CC]	1,02	,71	2,07	1	,14	2,79	,69	11,33
	[ERCC5(G/C) = GC]	-,16	,50	,10	1	,74	,84	,31	2,29
	[ERCC5(G/C) = GG]	0^b^	.	.	0	.	.	.	.
	[PY = 0]	-1,21	,88	1,87	1	,17	,29	,05	1,69
	[PY = 1-19]	-2,24	1,16	3,72	1	,05	,10	,01	1,03
	[PY ≥ 20]	0^b^	.	.	0	.	.	.	.

This model was obtained after adjustment to sex and age; a: The reference category is Pta tumor group; b: this parameter is set to zero because it is redundant; PY: pack years. Logistic regression: Number of observation = 193, Chi-square: 32.851, p = 0.035, Pseudo R-square = 0.087, Log likelihood = 187,948

**Table 5:** Effect of tobacco and *XPC*, *ERCC2 *and *ERCC5 *polymorphisms on pT1 tumors grade

								95% Confidence Interval for Exp(B)	
								
grade ^a^		B	Std. Error	Wald	df	**Sig**.	Exp(B)	Lower Bound	Upper Bound
III	Intercept	,38	3,21	,01	1	,91			
	Sex	-1,20	1,42	,72	1	,40	,30	,02	4,84
	Age	,55	,78	,49	1	,48	1,73	,37	8,05
	[XPC (A/C) = AA]	-,01	,82	,00	1	,98	,98	,20	4,90
	[XPC (A/C) = AC]	,14	,75	,03	1	,86	1,15	,26	4,98
	[XPC (A/C) = CC]	0^b^	.	.	0	.	.	.	.
	[ERCC2 (A/C) = AA]	,43	,92	,21	1	,64	1,53	,25	9,30
	[ERCC2 (A/C) = AC]	-,29	,97	,09	1	,76	,74	,11	4,96
	[ERCC2 (A/C) = CC]	0^b^	.	.	0	.	.	.	.
	[ERCC5 (G/C) = CC]	,58	,89	,42	1	,52	1,78	,31	10,26
	[ERCC5 (G/C) = GC]	-,08	,58	,02	1	,89	,92	,30	2,88
	[ERCC5 (G/C) = GG]	0^b^	.	.	0	.	.	.	.
	[PY = 0]	1,93	,82	5,54	1	,02	6,89	1,38	34,33
	[PY = 1-19]	-18,32	,00	.	1	.	1,10	1,10	1,10
	[PY ≥ 20]	0^b^	.	.	0	.	.	.	.

This model was obtained after adjustment to sex and age; a: The reference category is pT1 with Grade II tumor; b: this parameter is set to zero because it is redundant; PY: pack years. Logistic regression: Number of observation = 87, Chi-square: 24.173, p = 0.007, Pseudo R-square = 0.213, Log likelihood = 57.865

## Discussion

Bladder cancer is strongly related to occupational and environmental exposure to chemical carcinogens. Cigarette smoking is nevertheless responsible for more cases of bladder cancer than any other risk factors because of its high prevalence [3]. The damage caused by cigarette smoke is mainly removed by the nucleotide- excision repair pathway (NER), and to a lesser extent, the base-excision repair pathway [7]. In this population-based case-control study we investigated the effect of tobacco and gene repair polymorphisms on bladder cancer development and their association with tumors stage and grade.

By using logistic regression test we have found that non smokers and light smokers (1-19PY) were protected against bladder cancer development. This result confirms the idea which considered tobacco as the most important exogenous risk factor for bladder cancer [3]. Tobacco components, such as 4-aminobiphenyl (4-ABP), increase bladder cancer risk by inducing local somatic mutations. Indeed, the study of Feng et al. [21] has reported that cigarette smoke generates a substantial amount of 4-ABP and metabolically activated 4-ABP preferentially binds to codons 280 and 285 of the p53 gene. Moreover, Ouerhani et al. [22] have reported that the p53 and FGFR3 spectral mutations, which were the most frequent mutated genes in bladder cancer, appears to depend to the intensity of tobacco use (PY). When we studied the association between tobacco and tumors stages our data have reported that the risk of developing pT1 tumors decrease significantly on light smokers (p = 0.036, OR = 0.154; CI 95% = 0.027-0.882). This result confirms the study of Thompson et al. [23] who have suggested that bladder tumors in patients who smoke tend to be large, multifocal and demonstrate high-histological stage. In contrast, we have found that non-smoker patients with pT1 have at a significant 6.88-fold increased risk of developing a GIII grade compared to the reference group. Our hypothesis suggests that tobacco is not implicated on the determination of the tumors grade.

The alleles frequencies for XPC*C, ERCC2*C and ERCC5*C, in control group were estimated at 0.352, 0.331 and 0.326 respectively. These frequencies were different to which reported for the Caucasian populations. Indeed, Goode et al. [24] have reported the frequencies of 0.38 and 0.23 for the ERCC2*C and ERCC5*C variants. In fact Mechanic et al. [25] and Agalliu et al. [26] have reported that the frequencies of XPC*C, ERCC2*C and ERCC5*C, in control group were respectively estimated at 0.39/0.48, 0.36 and 0.23/0.45. The comparison of patients and controls according to XPC A > C polymorphism have showed that patients harboring XPC C/C genotype was associated with a significant 2.09-fold increased risk of developing bladder cancer compared to the control carrying the wild genotype. This results confirm the recent study of de Verdier et al. [27] who have found an association with the K939Q (A > C) polymorphism and bladder-cancer. This association is explained by the fact that Xeroderma pigmentosum type C (XPC), a NER gene, is considered as an important DNA damage recognition protein that binds to damaged DNA at a very early stage during DNA repair binds [9]. The NER pathway mainly removes bulky DNA adducts, which are typically generated from exposure to polycyclic aromatic hydrocarbons in tobacco smoke. Therefore, the NER pathway would be expected to play a more significant role in repairing tobacco carcinogen-induced DNA damage, whereas the other DNA-repair pathways play a less prominent role. However, although this polymorphism replaces the basic, hydrophilic amino acid lysine with the neutral amino acid glutamine, the risk association, as well as the functional consequences, remain controversial [28,29].

The comparison of patients and controls according to the frequencies of ERCC2 genotypes does not show a significant statistic difference. Our observation of no association of the *XPD *Lys751Gln genotypes with the risk of Bladder cancer is compatible with the findings from Shen et al. [13] and Gangwar et al. [30] who reported no association of the *ERCC2 *codon 751 polymorphism with bladder cancer risk. However, Gao et al.[16] and Li et al. [17] suggest that individuals who have the ERCC2 751Gln allele may be at an increased risk for bladder cancer. Authors explain this association by the fact that ERCC2 gene mutations can diminish the activity of TFIIH complexes giving rise to repair defects, transcription defects, and abnormal responses to apoptosis [31]. With regarding the ERCC5 Asp1104His polymorphism we confirm the study of Sanyal et al. [15] whom don't found a significant difference for genotype distributions between the bladder cancer cases and the controls.

The comparison of patients according to the clinical characteristics does not show a significant association between XPC, ERCC2 or ERCC5 polymorphisms and tumors stage and grade. Our results is compatible with the findings from Gangwar et al. [30] who reported no association of the *ERCC2 *codon 751 polymorphism with any stage and grade However, Chen et al. [12] have reported strong correlation of XPC deficiency, and the degree of malignancy of bladder tumors. This association is explained by the great correlation between XPC deficiency and p53 mutations. Indeed, the p53 alterations occur predominantly in invasive and high-grade superficial tumors [32]. Moreover Sakano et al. [33] have reported that the Asp1104His polymorphism in exon 15 of *ERCC5 *(rs17655) was associated with tumour stage and grade at presentation, especially in the subgroups divided by age. This association is explained by the fact that the Asp1104His polymorphism which was located in the *ERCC5 *C-terminus, is required for interactions with the transcription factor TFIIH in the incision complex of NER.

Although some of the results presented here are novel, this study has some limitations. Firstly, the sample size is small, limiting the precision of the statistical analyses. Secondly, the correlation between XPC C/C genotype and cell cycle gene alterations (such as p53) was not done. This correlation is very interesting to understand the mechanism of action of XPC on bladder cancer initiation. Besides that in the future, enlargement of sample sizes in the Tunisian population and analysis of somatic altered genes (which is already ongoing) will be essential to assess the role that environmental factors together with the genetic factors play as predictors of differential susceptibility to the presentation of malignancy.

## Conclusion

In this study we find statistical evidence from generalized ordered logistic models that non smokers and light smoker patients (1-19PY) are protected against bladder cancer development and invasiveness. Our results also indicate that patients with XPC C/C genotype were at increased risk for developing bladder cancer. Finally, we have reported that the SNP in the XPC, ERCC2 and ERCC5 genes don't affect the bladder tumors phenotype.

## Competing interests

The authors declare that they have no competing interests.

## Authors' contributions

KR carried out the experimental studies and drafted and completed the manuscript. IBB, KB, RM, NS and KH participated in the design of the study. MC, MRB, MS, FBO and MC participated in the tissue collection and tumor pathological characterisation. ABG participated in the design and coordination. SO conceived of the study, performed the statistical analysis and participated in the design and coordination as well as helped to draft the manuscript. All authors read and approved the final manuscript.

## Pre-publication history

The pre-publication history for this paper can be accessed here:

http://www.biomedcentral.com/1471-2407/11/101/prepub
